# Loss in Executive Functioning Best Explains Changes in Pain Responsiveness in Patients with Dementia-Related Cognitive Decline

**DOI:** 10.1155/2015/878157

**Published:** 2015-12-14

**Authors:** Miriam Kunz, Veit Mylius, Karsten Schepelmann, Stefan Lautenbacher

**Affiliations:** ^1^Gerontology Section, Department of General Practice, University Medical Center Groningen, University of Groningen, 9700 Groningen, Netherlands; ^2^Department of Neurology, Philipps University Marburg, 35043 Marburg, Germany; ^3^Department of Neurology, Helios Klinikum Schleswig, 24837 Schleswig, Germany; ^4^Physiological Psychology, Otto-Friedrich University Bamberg, 96045 Bamberg, Germany

## Abstract

There is ample evidence that dementia changes the processing of pain. However, it is not known whether this change in pain processing is related to the general decline in cognitive functioning or whether it may be related to specific domains of cognitive functioning. With the present study we tried to answer this question. We assessed different cognitive domains (orientation, memory, abstract thinking/executive function, aphasia and apraxia, and information processing speed) in 70 older patients with cognitive impairment (mild cognitive impairment up to moderate degrees of dementia). Pain responsiveness was assessed by measuring the nociceptive flexion reflex (NFR) threshold and facial responses to noxious electrical stimulation. Using regression analyses, we assessed which domain of cognitive functioning best predicted variance in pain responsiveness. Variance in pain responsiveness (NFR and facial expressions) was best explained by those items assessing executive functioning even when controlling for overall cognitive performance and memory functioning. The close association between executive functioning and pain responsiveness suggests that dementia-related neurodegeneration in prefrontal areas might result not only in reduced executive functioning but also in a loss of pain inhibitory potency, rendering the patient more vulnerable to pain. Our findings also suggest that pain assessment in dementia should be regularly completed by tests of cognitive functions.

## 1. Introduction

Prevalence rates for both dementia and pain are increasing with age. Given the demographic changes in the coming decades and the increasingly aging population there will be a substantial growth in the number of individuals who will be suffering from pain as well as from dementia [1]. When an individual is suffering from both pain and dementia, it is likely that these two conditions do not only cooccur but also will interact with each other, with dementia changing the processing of pain [2].

When trying to explain this interaction from a neurobiological perspective one could argue that there is a large overlap in brain areas involved in pain processing and in certain cognitive processes and that several of these areas are affected by dementia-related neurodegeneration [3]. For example, Alzheimer's disease seems to mostly affect the medial pain system, which is functionally responsible for the affective-motivational dimension of pain processing, with a focus on disruption in medial-temporal cortex areas [3]. This disruption in medial-temporal cortex areas could affect pain processing [3] as well as memory functioning [4], with both pain and memory functions possibly showing a parallel decline in the course of dementia. However, there is also ample evidence that prefrontal cortex areas deteriorate in the course of dementia. The prefrontal cortex is involved not only in descending pain inhibitory systems [5, 6] but also in executive functioning [7]. Thus, functional disruption of prefrontal areas might lead to a parallel worsening of pain modulation and executive functions in the course of dementia. While these neurobiological arguments alone might appear speculative, an interaction between pain and cognition can be corroborated from a neuropsychological perspective. Memory for pain can help avoid future contact with noxious stimuli or help successfully cope with pain, based on previous experience. However, it is also conceivable that action preparation and monitoring (precisely adjusted to the actual situation) are necessary to lower the noxious risk and to promote protective as well as coping behavior. Such planning of action is part of what is called “executive functions.”

So, which cognitive domain is most closely linked to previously described [8, 9] changes in pain responses in patients with dementia-related cognitive decline? Does memory functioning or executive functioning or maybe even other types of cognitive functioning explain dementia-related changes in pain processing? To assess the relative contribution of different cognitive functions to the prediction of pain processing in patients with dementia-related cognitive decline, we studied patients with manifest (mainly Alzheimer's disease or mixed forms of dementia) and potentially early forms (MCI) of dementia. For inclusion, patients had to show impairments in cognitive functioning with a preponderant decline in memory. To assess the general cognitive status as well as different cognitive domains at once, we used a comprehensive but not too demanding cognitive test battery called Structured Interview for the Diagnosis of Dementia of the Alzheimer Type, Multi-infarct Dementia and Dementia of other Etiology according to DSM-III-R, DSM-IV, and ICD-10 (SIDAM) [10]. The advantage of the SIDAM compared to the Mini Mental State Examination (MMSE) [11] is the inclusion of further items, which improves the resolution and distribution of the performance parameters. Cognitive functions included are “orientation,” “aphasia,” “apraxia,” “memory,” and “intellectual abilities/executive function,” with latter two functions being of special interest for the present study. We also used a version of the Trail Making Test A (Zahlen-Verbindungs-Test-G [12]) to assess “information processing speed.”

The two questions to be answered were as follows. (i) Which cognitive domain is most predictive of pain processing in patients with dementia-related cognitive decline? (ii) Does this most predictive cognitive domain add additional explained variance beyond that already explained by the general cognitive status (MMSE) of the patient?

## 2. Materials and Methods

### 2.1. Participants

Seventy participants over the age of 65 (mean age: 75.6 ± 7.0 years; ♀: 40, ♂: 30) with different degrees of dementia-related cognitive decline were included in the study. Participants were recruited amongst students of a local adult education center (Volkshochschule) (mostly those with mild cognitive impairments) and amongst inpatients from the Department of Neurology and the Department of Psychiatry and Psychotherapy of the University of Marburg (mostly those participants at slight and moderate stages of dementia). None had taken any analgesic medication for at least 24 hours prior to the test session. Participants with any condition other than cognitive impairment that could affect pain perception and pain report such as diabetes, hypertension, peripheral and central neuropathy, and neurological and psychiatric disorders were excluded from the study. Prior to the experiment, a thorough neurological examination (including examination of the sensory system, motor functioning (e.g., strength, gait, and deep tendon reflexes), autonomic testing, and sural neurography) was conducted in order to identify persons who met the exclusion criteria. We only included individuals with either dementia or mild cognitive impairment (MCI). MCI was diagnosed according to the criteria of Petersen et al. [13]. Based on these criteria, 35 elderly individuals were categorized as individuals with MCI (mean age: 75.4; mean MMSE: 25.9). Furthermore, 35 elderly individuals were categorized as patients with putative dementia (mean age: 75.7; mean MMSE: 16.4) and were examined by a neurologist or psychiatrist and diagnosed according to the criteria specified by the ICD-10, NINCDS-ADRDA [14], and NINDS-AIREN [15]. According to these diagnostic guidelines, analyses of cerebrospinal fluid, blood chemistry analysis, EEG, CT, or MRT and ECG analyses were conducted besides clinical assessment. Twenty-three patients met the NINCDS-ADRDA criteria for the clinical diagnosis of probable Alzheimer's disease or Mixed Dementia and 12 patients met the NINDS-AIREN criteria for the clinical diagnosis of probable Vascular Dementia.

The study protocol was approved by the ethics committee of the medical faculty of the University of Marburg. We took care that only patients with dementia who still had legal capacity were included in the study. After being informed in a slow and simple fashion, which was adjusted to the individual intellectual capacities, subjects gave written informed consent. We also provided instructions during testing as simple as possible and monitored the patients continuously for any signs of undue discomfort (verbally or nonverbally), in which case we stopped testing immediately.

### 2.2. Materials and Procedure

All testing was conducted during the hours of 3.00 p.m.–6.30 p.m. and it lasted for approximately 2 hours. Within these 2 hours, (i) a neuropsychological examination lasting approximately 30 minutes and (ii) pain responses to electrical stimulation (30 min) were assessed. We also conducted a neurological examination (as mentioned in the subject description) and assessed facial responses to pressure stimuli (the results have been reported elsewhere [8]). In order to help building up a nonthreatening atmosphere for the subjects, we decided to always start with pressure stimulation, since this procedure (for a detailed description, see Kunz et al. [8]) was much less likely to elicit anxiety or threat compared to electrical stimulation.

#### 2.2.1. Neuropsychological Examination

We used the Structured Interview for the Diagnosis of Dementia of the Alzheimer Type, Multi-infarct Dementia and Dementia of other Etiology according to DSM-III-R, DSM-IV, and ICD-10 (SIDAM) [10] as well as the Zahlen-Verbindungs-Test (ZVT) [12], which is similar to the Trail Making Test (version A) to assess different domains of cognitive functioning. The SIDAM is a mixed semistructured and structured instrument which has been developed for diagnosing mild cognitive impairment (MCI) and dementia. The cognitive section of the SIDAM consists of 55 questions and allows calculating subscores for the domains “orientation” (orientation for time and place), “memory” (immediate recall and short-term and long-term memory), “intellectual abilities/executive function” (abstract thinking and making plausibility judgments), and “aphasia and apraxia” (naming objects and performing according to instructions). The SIDAM also includes the Mini Mental State Examination. With the ZVT we assessed “information processing speed”.

#### 2.2.2. Pain Responsiveness

Given that we previously found that dementia was associated with changes in the nociceptive flexion reflex (NFR) and in facial responses [9] we focused on these two types of responses in the present study, although we also assessed self-report ratings and autonomic responses (see Kunz et al. [9] for more details).

*Assessment of the Nociceptive Flexion Reflex (NFR)*. Electrical stimulation and the NFR assessment (EMG recording) were performed using a standard electrodiagnostic device (Viking IV D, VIASYS Healthcare) with modified software. During electrical stimulation, the subjects were seated upright in a comfortable armchair with knees flexed at 130°. The stimulating electrode (bar electrode) was attached on the left calf over the pathway of the sural nerve (sural neurography was performed during the neurological examination preceding the testing in order to exclude patients with sensory polyneuropathy and in order to localize the sural nerve for NFR stimulation). This individualized procedure, in contrast to standardized retromalleolar stimulation in previous studies [16], allows exact stimulation of the sural nerve as determined during prior sural neurography. For recording, the differential surface electrode was attached ipsilaterally over the short head of the biceps femoris muscle with the reference electrode fixed near the tendon of the biceps femoris muscle at the head of the fibula bone. We inspected, cleaned, and abraded skin before to avoid electrode contact with skin abnormalities and to keep the impedance at the lowest level possible.

A time window of 80–150 ms was selected for the onset of the reflex in order to exclude early RII responses and voluntary limb movements according to the results of previous studies [17]. Furthermore, amplitude of at least 40 *μ*V within 100 ms after the reflex onset was required to reliably distinguish reflex responses from baseline fluctuations. A train of five impulses with 1 ms duration at a frequency of 250 Hz was used for stimulation. Between each stimulus a variable interval from 20 to 30 seconds was used in order to avoid habituation. The* NFR threshold* was assessed using the up-down staircase method. Stimulation intensity was increased in 3 mA increments until the flexion reflex RIII component was detected the first time or a maximum stimulus intensity of 40 mA was reached. Next, we lowered stimulus intensity in 2 mA steps until the reflex disappeared. After that, steps of 1 mA were used and the procedure was repeated until the reflex appeared and subsided two more times. Mean values of three peaks (current intensity that just elicited a reflex) and three troughs (current intensity that just no longer elicited a reflex) determined the reflex threshold. 

*Facial Responses*. After the NFR threshold was assessed, facial responses to 10 electrical stimuli with an intensity of 5 mA above the individual NFR threshold were assessed. We used suprathreshold intensities to ensure noxious stimulation levels. Facial responses were assessed and analyzed according to procedures described in detail previously [8, 9]. In short, the face of the subject was videotaped throughout the entire session and facial responses were later analyzed using the Facial Action Coding System (FACS) [18]. The FACS is a fine-grained anatomically based system which is considered the gold standard when decoding facial expressions, including the facial expression of pain. A certified FACS coder identified the frequency and the intensity (5-point scale) of the different Action Units. A software designed for the analysis of observational data (the Observer Video-Pro; Noldus Information Technology) was used to segment the videos and to enter the FACS codes into a time-related database. Time segments of 5 seconds after stimulus onset were selected for scoring. For purpose of necessary data reduction, we combined those AUs that represent facial movements of the same muscle as has been done in preceding studies without any loss of information [19–22].

To select those AUs that appeared to be pain-relevant in the present experimental context and to summarize these facial responses to composite scores, several steps were necessary: (1) AUs had to occur in more than 5% of the suprathreshold trials and (2) AUs had to be more frequent during suprathreshold trials than during those trials below the NFR threshold (those were taken from the NFR threshold assessment) (effect size *d* ≥ 0.5; these AUs are in italic font in Table 1). Following this, mean AU frequency and mean AU intensity values of the selected AUs were combined (product terms) to form a* composite score* of pain-relevant facial responses [9]. Due to the fact that the composite score was not distributed normally, scores were square root transformed.

### 2.3. Statistical Analysis

In order to find out which domain of cognitive functioning is most closely related to pain responsiveness in individuals with dementia-related cognitive decline, linear regression analyses were computed.

*Step 1 (forward selection).* The aim of Step 1 was to select out of the different domains of cognitive functioning those domains that contribute most to the prediction of variances in pain responsiveness. Thus, multiple regression analyses with forward selection were conducted separately for 2 criterion measures (NFR threshold and facial responses to pain). As predictors we entered the SIDAM subscores for the domains “orientation,” “memory,” “intellectual abilities/executive function,” and “aphasia and apraxia.” In addition “information processing speed” (based on the ZVT) was also entered.

*Step  2a (predictive power compared to MMSE).* The aim of Step 2a was to evaluate whether those domains that proved to be most predictive in Step 1 are indeed significantly better predictors of pain responsiveness than a general indicator of cognitive functioning. Therefore, we conducted blockwise multiple regression analyses. We entered the MMSE score into the first block of the regression analyses. In the second block, we entered the “best predictors of Step 1.” Regression analyses were again conducted separately for the two criterion measures.

*Step  2b (predictive power compared to memory functioning).* Impairment in memory functioning is the most prevalent cognitive impairment in individuals with MCI and dementia. In case the best predictors from Step 1 do not include the domain “memory,” the aim of Step 2b was to evaluate whether those domains that proved to be most predictive in Step 1 are indeed significantly better predictors of variances in pain responsiveness than the domain “memory.” Therefore, we conducted blockwise multiple regression analyses, entering “memory” performance into the first block and the “best predictors of Step 1” into the second block of the regression analyses. Regression analyses were again conducted separately for the two criterion measures.

For description of simple relationships between the predictor variables, Pearson's correlation coefficients were computed. Findings were always considered to be statistically significant at *α* < 0.05. SPSS 23 was used for all analyses.

## 3. Results

Table 2 gives an overview of the cognitive performance within the different cognitive domains assessed in the present sample. As can be seen, the average score of the MMSE was 21, suggesting mild dementia-related impairment overall. We also computed intercorrelations between the different domains of cognitive functioning. As expected, the different domains of cognitive functioning were all highly intercorrelated, with correlation coefficients ranging between *r*-values of .563 to values of .739. The descriptive data of pain responsiveness are also given in Table 2.

### 3.1. Which Domain of Cognitive Functioning Best Predicts Variance in Pain Responsiveness?

*Step 1 (forward selection).* Both linear regression analyses conducted showed that based on the forward selection procedure only the cognitive domain “intellectual abilities/executive function” remained a significant predictor in the model, whereas all other domains were excluded (see Table 3). The domain “intellectual abilities/executive function” explained about 16% of the variance in pain responsiveness.

*Step 2 (predictive power of the domain “intellectual abilities/executive function” compared to (a) MMSE and (b) memory functioning).* Using blockwise regression analyses entering (a) MMSE scores in the first block of the regression model, we found a significant increase in explained variance after entering “intellectual abilities/executive function” in the second block. This was true for both types of pain responses (NFR thresholds and facial responses). As can be seen in Table 4, “intellectual abilities/executive function” added approximately 5–8% of explained variance to the explanatory power of the MMSE score. Thus, “intellectual abilities/executive function” helps explain variances in pain responses even above and beyond a general indicator of cognitive functioning.

Using blockwise regression analyses entering (b) memory functioning in the first block of the regression model, we also found a significant increase in explained variance after entering “intellectual abilities/executive function” in the second block. This was true for both types of pain responses (NFR thresholds and facial responses). As can be seen in Table 4, “intellectual abilities/executive function” added approximately 5–7% of explained variance to the explanatory power of memory functioning. Thus, “intellectual abilities/executive function” helps explain variances in pain responses even above and beyond those explained by memory functioning.

## 4. Discussion

There were two major findings of the present study. (i) Increasing levels of cognitive decline appeared to be associated with enhanced pain responses. This interpretation can be based on both indicators of pain processing studied, facial expression of pain and NFR-reflex, which are independent from introspection and self-report, making them especially robust for the use in patients with dementia. (ii) Intellectual abilities/executive functions contributed overproportionally to the explanation of the changes in pain responses.

The first finding adds to the frequent observations that dementia (mainly Alzheimer's disease) is associated with changes in pain processing [2]. There is, however, some controversy with regard to the direction of change. Whereas in most of the studies patients with dementia appeared to be more sensitive to pain [8, 9, 23], some other studies showed no [24, 25] or opposite effects [26]. Part of the controversy can be resolved by considering the methods applied to assess pain because the behavioral and physiological indicators favor the assumption of an enhanced pain processing [8, 9, 23], while the parameters based on subjective report show no differences [24, 25] or even dampened pain processing [26].

The more interesting finding is the second one; namely, the changes in pain processing were best explained by the executive function of the patients. Even when the general cognitive status and memory dysfunction in the patients with MCI and dementia were controlled for, executive functions explained extra variance in pain responses. The testing of executive functions contained items of abstract thinking (explaining the meaning of idiomatic expressions and semantic differences) and judgement (describing pictures representing actions (Luria thematic pictures) and plausibility judgements) [10]. It is widely acknowledged that abstract thinking and judgement are both key components of executive functioning [27, 28]. The worse these capacities of abstract thinking and judgment were, the stronger the increase in pain processing was. This fits well with the idea that the dorsolateral prefrontal cortex (DLPC) is responsible for both these higher cortical functions of cognition [29] and the cortical phase of the descending inhibitory control system of pain [30]. Thus, worsening of this type of executive functions and the loss of pain inhibitory potency might well run in parallel. The observation that pain activates again and again the DLPC in patients with Alzheimer's disease might accordingly be seen as unsuccessful attempts to start the cortical phase of descending pain inhibition because of a loss of DLPC functionality [31]. We have to acknowledge that no study so far has actually looked at brain activation patterns accompanying the abstract thinking and judgement tasks of the SIDAM, and thus we cannot be sure that these items were indeed linked to DLPFC dysfunction or to dysfunctions in other frontal areas. Therefore, the interpretation that DLPFC dysfunction underlies both the decline in executive functioning and the decline in pain inhibition is only very speculative.

A study by Scherder et al. [32] seems to corroborate the present finding of a close relation between pain processing and executive functioning by showing a significant association of executive functions (Digit Span Backward, Category Fluency, and Knox's Cube Imitation Test) and the intensity of arthrosis or arthritis pain in patients with Alzheimer's disease. However, the direction of the correlation was reversed. The better the executive functions were, the more the patients suffered from pain. The close but intricate relationship between disruption of prefrontal areas and pain processing is also indicated by Fletcher et al. [33] who assumed on one hand a higher pain prevalence in patients with frontal lobe damage but on the other hand assumed blunted responsiveness to pain in behavioral variant FTD and semantic dementia. This unpredictable closeness between prefrontal functions and pain is also nicely described by Denk et al. [30] who showed clearly that the whole prefrontal cortex appeared to be more or less engaged in certain stages of pain processing. (inhibition, reward, and perception). In line with this, Oosterman et al. [34] could show that executive function predicted pain sensitivity to a cold pressor test, with higher executive functioning being associated with less sensitivity to pain (the same direction that we found). In consequence, our finding that the disruption of executive functions in patients with mild-to-moderate degrees of dementia-related cognitive impairment is related to a deterioration of pain inhibition and, in consequence, to enhanced pain responses is very plausible. However, it may well be that the testing of other executive functions with implementation in other areas of the prefrontal cortex may change the picture substantially.

Our study has methodological strengths and weaknesses. The tests of pain responses were largely independent from self-report and were robust for the use with patients with dementia as shown in other studies [9]. The SIDAM is a comprehensive test battery for cognitive functioning tailored to the testing capability of patients with dementia. However, it includes only a few items for each cognitive domain, and how strongly the items assessing “abstract thinking” and “judgement” correlate with other tests of executive functioning has not been tested, which may lower its reliability and its validity, respectively.

## 5. Conclusions

The cognitive decline in patients with dementia-related cognitive impairment appeared to be paralleled by an enhancement of pain responses. This close relation between cognitive functions and pain parameters gives further support to the idea that pain assessment in patients with dementia should be regularly completed by tests of cognitive functions. Memory functions proved to be important for this relation but did not exceed other cognitive domains in this respect. In contrast, the disruption of executive functioning appeared to be especially predictive for enhanced pain responses in patients with dementia-related cognitive decline. Given the present results, we suggest that pain diagnostics in patients with dementia should be accompanied by an assessment of executive functions.

## Figures and Tables

**Table 1:** Facial Action Units (AUs) with a critical frequency of occurrence of more than 5% in supra-NFR threshold trials. Effect sizes for frequency differences between “below threshold” and “suprathreshold” trials are given.

Action Unit	Description	Frequency of occurrence	
		Percent^a^	Effect size (Cohen's *d*)
*AU4*	*Brow lower*	**32.0**	**d** = 1.05
*AU6/7*	*Orbit tightening*	**78.7**	**d** = 1.33
*AU9/10*	*Levator contraction*	**23.7**	**d** = 1.51
AU14	Dimpler	12.6	*d* = 0.42
*AU17*	*Chin raise*	**14.0**	**d** = 0.65
AU18	Lip pucker	11.3	*d* = 0.37
*AU25/26/27*	*Mouth opening*	**62.8**	**d** = 0.75
AU45	Blink	168.6	*d* = 0.19

Medium and strong effect sizes (*d* ≥ 0.5) are marked in bold.

^a^Percent denotes the percentage of occurrence in the entire suprathreshold segments.

**Table 2:** Descriptive data of the cognitive performance within the different domains of cognitive functioning assessed and of pain responsiveness.

			Descriptive data	
			Mean	SD
Cognitive functioning	SIDAM	Orientation	14.8	5.4
		Memory	8.6	4.2
		Intellectual abilities/executive function	3.3	1.7
		Aphasia and apraxia	7.6	2.0
	ZVT	Information processing speed	84.1	54.7
	SIDAM	MMSE	21.2	6.2

Pain responsiveness	NFR threshold (in mA)		16.4	9.0
	Facial response (FACS composite score, sqrt.)		2.6	1.7

**Table 3:** Linear regressions analyses (forward selection) predicting variances in pain responsiveness (NFR threshold and facial responses).

Significant predictors	Criterion: NRF threshold			Criterion: facial response		
	*β*	*r* ^2^	*p*	*β*	*r* ^2^	*p*
Intellectual abilities/executive function	.411	.169	<.001	−.393	.154	.001

Excluded						
orientation	.060		.663	.026		.852
Memory	.220		.129	−.058		.697
Apraxia and aphasia	.195		.177	−.137		.355
Information processing speed	−.112		.409	.088		.521

**Table 4:** Linear regressions analyses (blockwise) showing the predictive power of “intellectual abilities/executive function” to explain variance in pain responsiveness (NFR threshold and facial responses) after controlling for (a) the general cognitive functioning and (b) memory functioning.

Step	Predictor	Criterion: NRF threshold					Criterion: facial response				
		*β*	*t*	Total *r* ^2^	Δ*r* ^2^	*p* (Δ*r* ^2^)	*β*	*t*	Total *r* ^2^	Δ*r* ^2^	*p* (Δ*r* ^2^)
(a)											
1	MMSE	.356	3.136	.126	.126	**.003**	−.279	−2.378	.078	.078	**.020**
2	Intellectual abilities/executive function	.315	2.081	.179	.053	**.041**	−.377	−2.444	.154	.077	**.017**
(b)											
1	Memory	.394	3.533	.155	.155	**.001**	−.286	−2.443	.082	.082	**.017**
2	Intellectual abilities/executive function	.278	1.971	.204	.049	**.046**	−.355	−2.409	.156	.074	**.019**
